# Primary synovial sarcoma of parotid gland with intravenous extension into the heart

**DOI:** 10.1259/bjrcr.20200080

**Published:** 2020-07-02

**Authors:** Avinash D Gautam, Shweta Jain, M Ravi Sankar, Neeraj Jain

**Affiliations:** 1 Department of Radiodiagnois, SGPGIMS, Lucknow, Uttar Pradesh, India; 2 Department of Pathology, SGPGIMS, Lucknow, Uttar Pradesh, India; 3 Department of Neurootology, SGPGIMS, Lucknow, Uttar Pradesh, India; 4 Department of Radiodiagnosis, SGPGIMS, Lucknow, Uttar Pradesh, India

## Abstract

We are reporting a case of a 47-year-old male with primary synovial sarcoma of the right parotid gland with tumor thrombus extension in the right internal jugular vein and right atrium. The rarity of this occurrence as documented in the review of the literature provides for uncertainty about proper management. Our case represents a rare occurrence with the unique radiological finding that has implications for management.

## Introduction

Tumor thrombus within neck veins is an uncommon event in the head and neck malignancies, and it has most often been reported in association with thyroid malignancies.^1–7^ Here we report a case of primary synovial sarcoma of the parotid gland with direct i.v. thrombus extending down the right internal jugular vein into the right atrium, evident in the form of a filling defect with the expansion of right internal jugular vein documented with CT.

Our case is a probably first case report, which shows the direct i.v. extension of parotid tumor into the right atrium.

## Case report

A 47-year-old male with no smoking, drinking, or no history of previous malignancy was initially referred to the department of radiodiagnosis for characterization of a growing painless right parotid mass that had been present for 1 year with rapid progression within three months.

**Figure 1.**
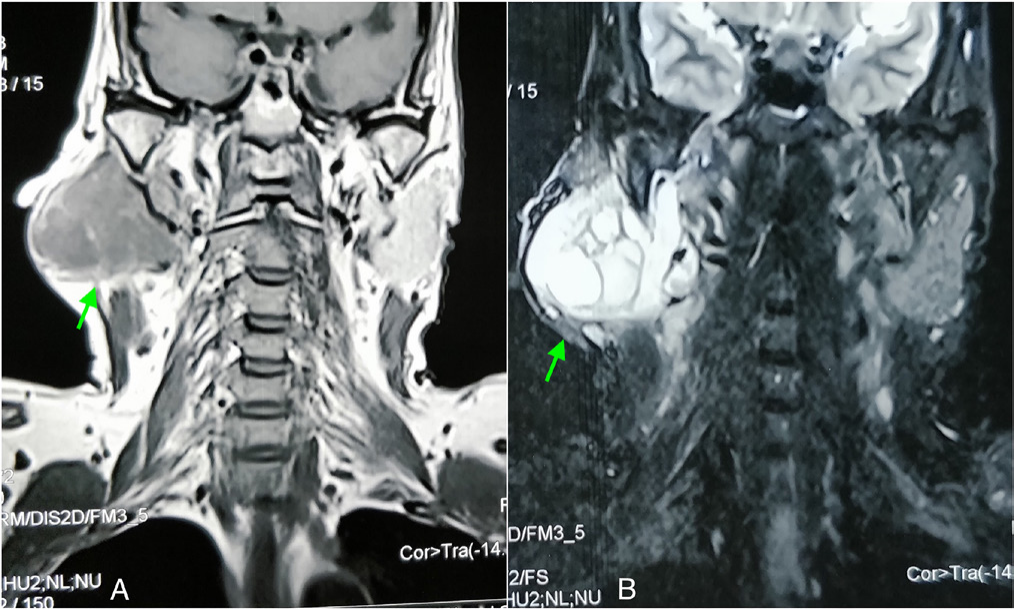
T1 (A) and T2 fat sat (B) coronal MRI images shows a lobulated well-circumscribed mass lesion involving both superficial and deep lobe of right parotid gland. The lesion shows hypointense signal on T1 and bright signal on T2.

Six month before presenting to our institution, the patient had noticed a right neck mass for which MRI was done outside, which showed a well-circumscribed, heterogeneous mass with low signal intensity on T1W images and high intensity on T2W images, involving both superficial and deep lobe of the right parotid gland, closely abutting right internal carotid artery and right internal jugular vein and extending into the right para-pharyngeal space with no evidence of tumor thrombus noted at that time (Figure 1).

Upon presentation at our institution, physical examination demonstrated significant enlargement in the mass’s size with no evidence of palpable veins, or tenderness in the neck. However, there was a history of rapid growth within 3 months. With these clinical findings, the patient underwent a CT scan, which demonstrated a fairly large (6.2×4.4 cm) heterogeneously enhancing mass lesion with internal necrotic changes arising from the right parotid gland and involving both superficial and deep lobe with perilesional fat stranding.

**Figure 2.**
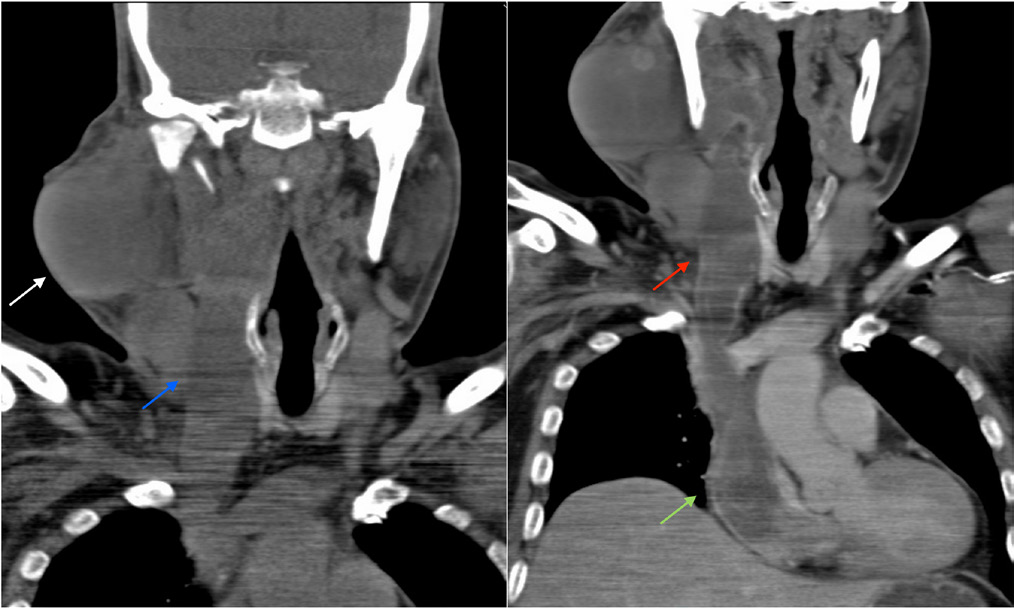
NCCT (A) coronal image shows well-circumscribed lobulated isodense mass lesion arising from right parotid gland (white arrow) with dilated right internal jugular vein (blue arrow). CECT (B) coronal image shows parotid lesion extend in right internal jugular vein (red arrow) which appears distended and further down into right atrium (green arrow).

On further evaluation, mass is seen to extend into the right internal jugular vein, which appears expanded and showed enhancing intraluminal filling defect on post-contrast images suggestive of tumor thrombus into the vessel (Figure 2). The tumor thrombus extends further down into the right atrium. Based on the imaging findings, the possibility of the malignant lesion was suggested.

**Figure 3.**
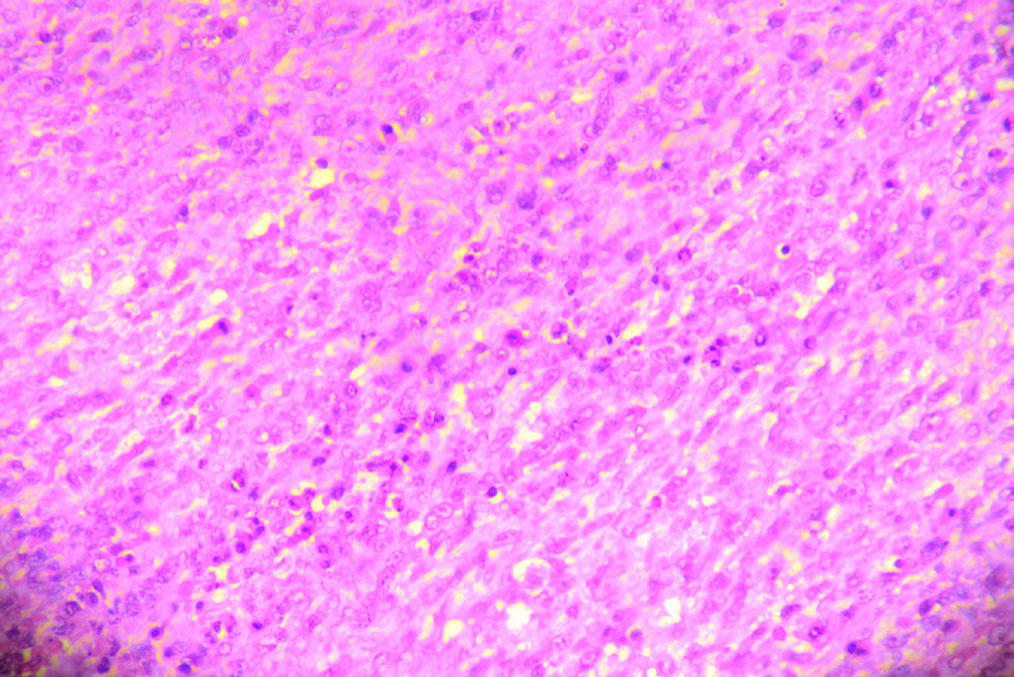
The section shows a tumor composed of sheets of round to spindle cells. These cells show vesicular nuclear chromatin, prominent nucleoli, ill-defined cytoplasm, and brisk mitotic figures.

Subsequently, histopathological analysis revealed a tumor composed of sheets of round to spindle-shaped cells with large areas of necrosis with cells showing vesicular nuclear chromatin, prominent nucleoli, ill-defined cytoplasm, and brisk mitotic figures. On immunohistochemistry, cells were positive for vimentin, suggestive of a malignant mesenchymal tumor. The final histopathology report was suggestive of monomorphic synovial sarcoma (Figure 3).

Due to the advanced stage of the tumor, radiotherapy followed by chemotherapy was planned. However, the patient died two months after the treatment was planned owing to the severity of the disease.

## Discussion

Parotid gland tumors are not uncommon, the majority of them are benign. Pleomorphical adenoma is the most common benign salivary gland tumor in adults (70–80% of all salivary gland benign tumors) with parotid gland involvement is the most common. Malignant lesions of the parotid gland are uncommon seen in around 20% of cases. Mucoepidermoid carcinoma (8–15%) is the most common type of parotid malignancy, followed by adenoid cystic carcinoma (5%) and acinic cell carcinoma, other uncommon parotid malignancies include primary adenocarcinoma, salivary duct carcinoma, primary squamous cell carcinoma, lymphoma, and synovial sarcoma.^8^

Synovial sarcoma is predominantly located near the joints of lower limbs, particularly knee joint and also arises from tendon sheath and bursa, however contrary to its name, it is also reported to be originated from other nonsynovial sites. Synovial sarcoma of the parotid gland is an extremely rare condition with only a few case reports are available in the literature. The hypopharynx is the most common site in head and neck with the larynx being the least common. However, in their study Al-Daraji W et al. revealed that the parotid gland is commonly involved in head and neck region^9^ . Mono-phasic and bi-phasic are the two types of synovial sarcoma, the biphasic variety contains both spindle and epithelial cell, while mono-phasic type has spindle cells only. Mesenchymal cell or myoepithelial cell of terminal duct are believed to be cells of origin that undergoes synovioblastic differentiation^10^.

CT and MRI may be used to determine the site of origin, delineate tumor extension, detect lymphadenopathy, identify calcification, and to evaluate possible airway compromise. MRI because of its excellent soft-tissue resolution, is considered the investigation of choice for detecting and staging of soft-tissue tumors of head and neck. Both CT and MRI features of synovial sarcomas are nonspecific with no pathognomonic features were described in the published literature. The usual presentation is of a well-defined solid lesion with occasional cystic or hemorrhagic changes and calcification.

These lesions exhibit intermediate signal on T1 and heterogeneously hyperintense signal on T2W images, respectively.^11,12^ Because of their imaging features such as smooth margins and lack of invasive nature, they were frequently misclassified as benign lesions.^13^

A malignant lesion may be associated with thrombus formation in the adjoining vein, which may be a result of continuous extension from the primary mass or due to stasis of flow as a result of compression.

Differentiation of bland thrombus from malignant tumor thrombus is crucial as it affects the future course of management, such as the extent of surgical resection, radiotherapy planning, and prognosis. Bland thrombus appears homogeneous, does not show contrast enhancement, and a thrombosed vein is not disproportionately distended on CT scan. Similar to CT, on the MRI, the bland thrombus does not show enhancement and exhibits hypointense signal on T2W sequences. Malignant thrombus causes abnormal expansion of the venous lumen, adheres to the vessel wall, and shows continuity with the primary tumor. Malignant thrombus exhibit enhancement on both CT and MRI similar to the primary mass and shows intermediate to hyperintense signal on T2W sequences^14^. Few studies show the utility of diffusion-weighted MRI in differentiating bland from malignant thrombus depending upon ADC values. If the thrombus’ ADC value is similar to the primary tumor, the possibility of malignant thrombus is high likely^15,16^

Direct tumor extension of head and neck malignant tumor into an adjacent vein is rarely described in the literature. Few studies had described the association of thyroid cancer with thrombosis of the accompanied vein.^3,5,7,17,18^ The existence of a tumor thrombus in the internal jugular vein from thyroid cancer was first described in 1991.^4^ Since then there were reports of thrombosis of vein from a metastatic lesion of the parotid gland, tumors of the deep lobe of parotid causing thrombosis of the internal jugular vein and reports of acute parotitis causing thrombosis of the internal jugular vein.^7,19,20^ However, we could find only one case of a 91-year-old male patient with a parotid tumor associated with tumor thrombosis of the right external jugular vein.^21^ In our case, the thrombus was in the internal jugular vein extending down to the superior vena cava and subsequently right atrium. Other head and neck tumors have also been reported to invade or grow within the great vessels, among those, paraganglioma was the most common.^22,23^

## Learning points

1. Sarcoma of the parotid gland is a rare neoplasm whose presence should be suspect whenever there is an association of local invasion and i.v. extension.
2. Imaging findings of synovial sarcoma are non-specific, however the presence of venous extension on imaging may indicate malignant nature.
3. Intravenous and subsequent intracardiac extension should be actively sought in the presence of locally invasive parotid neoplasm as its presence or absence might determine the appropriate management.
4. CT and MRI are both useful modalities to diagnose and assess the extension of tumor thrombus. However, for proper assessment scan area should cover the entire extent of tumor extending from the base of the skull cranially to the junction of superior vena cava and right atrium.

## Conclusions

Since synovial sarcoma of the parotid gland represents a rare entity; the diagnosis and clinical management can be a challenge. As the CT and MRI findings are nonspecific, histopathological confirmation is always needed. Interestingly our case demonstrates the rare occurrence of tumor thrombus reaching up to right atrium along with synovial sarcoma of the parotid gland, which has not been reported in any of the earlier publications and hence needs to be taken care of in the evaluation of such cases.
